# Identification of frailty in primary care: accuracy of electronically derived measures

**DOI:** 10.3399/BJGP.2022.0574

**Published:** 2023-07-25

**Authors:** Karin MA Swart, Amber AWA van der Heijden, Marieke T Blom, Jetty A Overbeek, Giel Nijpels, Hein PJ van Hout, Petra JM Elders, Ron MC Herings

**Affiliations:** PHARMO Institute for Drug Outcomes Research, Utrecht, and Amsterdam UMC location Vrije Universiteit Amsterdam, General Practice, Amsterdam, the Netherlands.; Amsterdam UMC location Vrije Universiteit Amsterdam, General Practice, Amsterdam, and Amsterdam Public Health, Amsterdam, the Netherlands.; Amsterdam UMC location Vrije Universiteit Amsterdam, General Practice, Amsterdam, and Amsterdam Public Health, Amsterdam, the Netherlands.; PHARMO Institute for Drug Outcomes Research, Utrecht, and Amsterdam UMC location Vrije Universiteit Amsterdam, General Practice, Amsterdam, the Netherlands.; Amsterdam UMC location Vrije Universiteit Amsterdam, General Practice, Amsterdam, and Amsterdam Public Health, Amsterdam, the Netherlands.; Amsterdam UMC location Vrije Universiteit Amsterdam, General Practice, Amsterdam, and Amsterdam Public Health, Amsterdam, the Netherlands.; Amsterdam UMC location Vrije Universiteit Amsterdam, General Practice, Amsterdam, and Amsterdam Public Health, Amsterdam, the Netherlands.; PHARMO Institute for Drug Outcomes Research, Utrecht, and Amsterdam UMC location Vrije Universiteit Amsterdam, Epidemiology & Data Science, Amsterdam, the Netherlands.

**Keywords:** electronic medical records, frailty, primary health care

## Abstract

**Background:**

Routinely collected clinical data based on electronic medical records could be used to define frailty.

**Aim:**

To estimate the ability of four potential frailty measures that use electronic medical record data to identify older patients who were frail according to their GP.

**Design and setting:**

This retrospective cohort study used data from 36 GP practices in the Dutch PHARMO Data Network.

**Method:**

The measures were the Dutch Polypharmacy Index, Charlson Comorbidity Index (CCI), Chronic Disease Score (CDS), and Frailty Index. GPs’ clinical judgement of patients’ frailty status was considered the reference standard. Performance of the measures was assessed with the area under the receiver operating characteristic curve (AUC). Analyses were done in the total population and stratified by age and sex.

**Results:**

Of 31 511 patients aged ≥65 years, 3735 (11.9%) patients were classified as frail by their GP. The CCI showed the highest AUC (0.79, 95% confidence interval [CI] = 0.78 to 0.80), followed by the CDS (0.69, 95% CI = 0.68 to 0.70). Overall, the measures showed poorer performance in males and females aged ≥85 years than younger age groups (AUC 0.55–0.58 in females and 0.57–0.60 in males).

**Conclusion:**

This study showed that of four frailty measures based on electronic medical records in primary care only the CCI had an acceptable performance to assess frailty compared with frailty assessments done by professionals. In the youngest age groups diagnostic performance was acceptable for all measures. However, performance declined with older age and was least accurate in the oldest age group, thereby limiting the use in patients of most interest.

## INTRODUCTION

Frailty is a common condition at older ages, characterised by loss of biological reserves across multiple organ systems and vulnerability to physiological decompensation after a stressor event.^1^ Frailty is associated with poor health outcomes, including falls, disability, admission to hospital, and mortality.^2^^–^6 Given the increased numbers of older people with frail health, care models should include frailty to focus on optimising health and avoiding the admission to hospital of frail and well older adults alike. Detection of frail older people can support timely management to maintain or improve functioning.^7^ Screening tools, such as frailty scales, and an understanding of a patient’s cognitive condition, physical function, and functional reserve, might alert the physician to start frailty management.^8^

Many frailty measures have been developed to identify patients with frail health in clinical practice.^9^ The most commonly used method to identify frailty in research settings combines questionnaires and functional measures.^10^ Alternatively, frailty has been operationalised, among other measures, as a risk index by counting the number of impairments accumulated over time, including disability, diseases, physical and cognitive impairments, psychosocial risk factors, and geriatric syndromes.^11^ Furthermore, methods have been developed to use routinely collected clinical data based on electronic medical records to define frailty. A significant advantage of these measures for clinicians is that no additional data collection is needed. They can be easily applied, thereby increasing their applicability in research and care settings, and they might make the identification process of frail older people more efficient.

However, hardly any studies have validated frailty measures against a diagnostic reference standard such as clinical judgement. Most validations reported associations with future adverse events. Varying results regarding the strength of the associations with mortality might be caused by varying distributions of the age and sex of the validation populations.^12^ In addition, Clegg *et al* created categories from fit to severe frailty purely on statistical distribution in an adult population between 65 and 95 years of age. It is still not clear how this categorisation relates to the clinical judgement of professionals.^13^

**Table:** How this fits in

Routinely collected clinical data might aid healthcare professionals in identifying frail older people. However, there is a lack of studies that have validated frailty measures against a diagnostic reference standard such as clinical judgement. In this study, it was found that, among the four measures evaluated, only the Charlson Comorbidity Index had an acceptable level of performance for assessing frailty, regardless of age. Although all four measures can be used to identify frailty in young older people (65‒74 years), their performance declined with increasing age.

The current study aims to estimate and compare the ability of four potential frailty measures used in research and clinical practice that make use of electronic medical records to identify older patients who were actually considered frail according to their GP. The four measures are the Dutch Polypharmacy Index (DPI), the Charlson Comorbidity Index (CCI), the Chronic Disease Score (CDS), and the Frailty Index (FI). In addition, the study aimed to compare the diagnostic performance of these measures across sex and age groups. The hypothesis was that electronic medical records can be used to identify frail older people, and the diagnostic performance might differ across age groups.

## METHOD

### Study population

Data for this retrospective cohort study were obtained from 36 GP practices from the PHARMO Data Network in the Netherlands that routinely coded frailty as part of older care programmes in 2019.^14^ These practices served a total population of 31 511 patients aged ≥65 years. The electronic medical records of the GP practices include information on diagnoses and symptoms, laboratory test results, and referrals to specialists and healthcare product/drug prescriptions. Diagnoses and symptoms were coded according to the International Classification of Primary Care (ICPC),^15^ and prescription drugs were coded according to the World Health Organization Anatomical Therapeutic Chemical (ATC) Classification System.^16^

All data from patients and practices were anonymised.

### Reference standard

GPs’ clinical judgement of patients’ frailty status was considered the reference standard (ICPC diagnosis code A05, derived from episodes). Within the older care programme, no strict definition of frailty was used, as the group of frail older people is heterogeneous by definition. Instead, a pragmatic definition was applied with loss of autonomy as a core manifestation and starting point for frail older people. This was judged by the GP. The GP’s clinical judgement of frailty has been shown to be an accurate indicator of frailty and a strong predictor of future mortality and long-term care admission.^10^^,^^17^

### Frailty measures

Four measures used in research and clinical practice to distinguish patients who are frail from those who are not are the DPI, the CCI, the CDS, and the FI (Box 1). All four multimorbidity measures are widely used in epidemiological studies and, especially the DPI and FI, in clinical practice.^18^

**Box 1.** Global characteristics of the four frailty measures

**Characteristic**	**DPI**	**CCI**	**CDS**	**FI**
**Definition**	Regular use of ≥5 medicines	Sum of weighted morbidity scores, based on its mortality risk	Comorbidity score based on the aggregate number of prescription medications	Number of health deficits divided by the total number of 50 deficits
**Initial purpose**	To identify polypharmacy	To predict mortality risk attributable to comorbidity	To predict health outcomes	To predict adverse health outcomes in older people
**Setting where it has been developed**	Primary care	Hospital	Pharmacy	Primary care
**Updates**	NA	ICPC codes mapped to ICD codes	Including novel pharmacotherapies	NA
**Input data**	Medication records — number of ATC codes (third level)	Comorbidity records — ICPC codes for comorbidity conditions	Medication records, age, sex — ATC classes of medication for treatment of different chronic diseases	Health deficits — ICPC codes of 50 health deficits
**Setting in which it is mainly applied**	Research + clinical practice	Research	Research	Research + clinical practice

*ATC = Anatomical Therapeutic Chemical. CCI = Charlson Comorbidity Index. CDS = Chronic Disease Score. DPI = Dutch Polypharmacy Index. FI = Frailty Index. ICD = International Classification of Disease. ICPC = International Classification of Primary Care. NA = Not applicable.*

The DPI is based on the concurrent regular use of medications, based on medication prescriptions. It is defined as the concurrent regular use (at least three single prescriptions, including at least one prescription in the preceding 6 months) of five or more medicines.^19^ The use of several medicines within one pharmacological subgroup (ATC third level) is counted as one.

The CCI was initially developed to measure the risk of 1-year mortality attributable to comorbidity and is based on diagnoses registered in the GP medical records. The CCI included 19 conditions that are weighted based on the severity of the condition. The CCI is calculated by summation of the weighted comorbidity scores.^20^

The CDS is a comorbidity measure based on 1 year of medication prescription data and age and sex. Classes of medication are weighted to correspond to disease complexity and severity.^21^ The CDS was adapted by the research group to also include additional ATC codes of newly developed drugs to the medication classes.

The FI is based on a predefined list of 50 health deficits. The FI (range 0 to 1) is calculated by dividing the number of present deficits in a patient by all 50 deficits. The lookback period is 6 months (for instance, for mood symptoms) or 5 years (for instance, for fractures), depending on the clinical relevance.^22^

### Statistical analysis

The characteristics of the study population are presented for the total study population and stratified by age (65–74, 75–84, and ≥85 years). Categorical variables are presented as numbers and proportions, and continuous variables were presented as mean (standard deviation [SD]) or median (interquartile range [IQR]) based on their distributions. In the total population and in subgroups of age and sex the ability of the index to distinguish between patients who were frail and those who were not according to the GP was assessed by calculating the area under the receiver operating characteristic curve (AUC). An AUC was considered excellent for values between 0.9 and 1.0, good for values between 0.8 and 0.9, acceptable for values between 0.7 and 0.8, poor for values between 0.6 and 0.7, and failed for values between 0.5 and 0.6.

The sensitivity, specificity, positive predictive value (PPV), and negative predictive value (NPV) were calculated for each index. The optimal cut-off values for identifying the frailty of each index within each subgroup were based on the Youden index (sensitivity + specificity −1) maximising the sum of sensitivity and specificity of each index with an equal weight of the two measures. Calibration (that is agreement between predicted and observed frailty incidence) was assessed by visual inspection of calibration plots and the observed and expected frailty incidence ratio. Calibration plots were created by plotting the observed mean incidence of frailty against the expected mean frailty incidence within deciles of the predicted probability of frailty.

Differences between GPs in their opinion on frailty may result in discrepancies in the assignment of frailty status in patients with similar comorbidity profiles but in different GP practices. To take these potential differences in frailty assignment into account, a sensitivity analysis was performed by testing the performance of the measures stratified by GP practice. Moreover, the performance of the measures was tested in the subgroup of GPs with an age- and sex-standardised frailty prevalence within the IQR of the total population. All analyses were performed using R (version 4.2.2).

As the underlying data represent attended medical care, it was assumed that the absence of a record meant no occurrence, for example, if an indicator of disease was missing for a patient, it was assumed that the patient did not have the disease.

## RESULTS

Of the total study population of 31 511 patients (mean age 75.0 years, 45.9% males), 3735 (11.9%) patients were classified as frail by their GP (Table 1). As expected, with increasing age groups a higher proportion of the people was classified as frail by their GP (Table 1).

**Table 1.** Characteristics of the study population, according to age

**Characteristic**	**Total (*n* = 31 511)**	**Age 65–74 years (*n* = 17 200)**	**Age 75–84 years (*n* = 10 138)**	**Age ≥85 years (*n* = 4173)**
**Age, years, mean (SD)**	75.0 (7.6)	69.3 (2.8)	79.1 (2.8)	88.9 (3.6)
**Males, *n* (%)**	14 472 (45.9)	8422 (49.0)	4529 (44.7)	1521 (36.4)
**Contacts with the GP in last year, median (IQR)**	6 (2–12)	5 (2–9)	7 (3–13)	12 (5–21)
**Home visits by the GP in last year, median (IQR)**	0 (0–0)	0 (0–0)	0 (0–1)	2 (0–6)
**Consultation gap in days,^a^ median (IQR)**	33 (6–99)	40 (7–112)	31 (6–91)	17 (3–56)
**Number of chronic medications,^b^ mean (SD)**	4.6 (3.8)	3.4 (3.5)	5.3 (3.9)	6.1 (4.1)
**Frail according to the GP, *n* (%)**	3735 (11.9)	355 (2.1)	1478 (14.6)	1902 (45.6)
**DPI (≥ 5 medicines), *n* (%)**	14 294 (45.4)	6166 (35.8)	5470 (54.0)	2658 (63.7)
**CCI, mean (SD)**	5.1 (2.3)	4.1 (1.8)	5.9 (2.1)	7.2 (2.2)
**CDS, mean (SD)**	5.6 (4.2)	4.8 (1.4)	6.4 (4.2)	7.0 (4.1)
**FI, mean (SD)**	0.23 (0.12)	0.21 (0.12)	0.26 (0.12)	0.27 (0.13)

a *Number of days between the reference date and last contact date.*

b *Number of medicines prescribed at least three times in the past year of which at least one prescription in last 6 months. CCI = Charlson Comorbidity Index. CDS = Chronic Disease Score. DPI = Dutch Polypharmacy Index. FI = Frailty Index. IQR = Interquartile range. SD = standard deviation.*

### Frailty measures in total study population

In the total study population, the CCI showed the highest AUC (0.79, 95% confidence interval [CI] = 0.78 to 0.80) followed by the CDS (0.69, 95% CI = 0.68 to 0.70) (Table 2). Sensitivity to identify frailty was highest when using the DPI or the CCI (0.72, 95% CI = 0.71 to 0.73 and 0.74, 95% CI = 0.73 to 0.76, respectively). Specificity was highest for the CCI (0.70, 95% CI = 0.69 to 0.70). Calibration plots of the four measures are presented in Supplementary Figure S1. There was a good agreement between predicted incidence and observed incidence in different deciles of the predicted probability of frailty of the four measures.

**Table 2.** Diagnostic performance of four measures to identify frailty^a^

**Measure**	**AUC**	**Cut-off**	**Sensitivity**	**Specificity**	**PPV**	**NPV**
**DPI**	0.65 (0.64–0.66)	1	0.72 (0.71–0.73)	0.58 (0.58–0.59)	0.19 (0.18–0.19)	0.94 (0.94–0.94)
**CCI**	0.79 (0.78–0.80)	6	0.74 (0.73–0.76)	0.70 (0.69–0.70)	0.25 (0.24–0.25)	0.95 (0.95–0.96)
**CDS**	0.69 (0.68–0.70)	7	0.64 (0.63–0.66)	0.63 (0.62–0.63)	0.19 (0.18–0.20)	0.93 (0.93–0.93)
**FI**	0.66 (0.65–0.67)	0.28	0.62 (0.60–0.64)	0.65 (0.65–0.66)	0.19 (0.19–0.20)	0.93 (0.92–0.93)

a *Cut-offs for identifying frailty (patients were considered frail above the cut-off) were based on the most optimal predicted probability estimated by the Youden index. AUC = area under the receiver operating characteristic curve. CCI = Charlson Comorbidity Index. CDS = Chronic Disease Score. DPI = Dutch Polypharmacy Index. FI = Frailty Index. NPV = negative predictive value. PPV = positive predictive value.*

### Frailty measures in subgroups of age and sex

**Figure 1.**
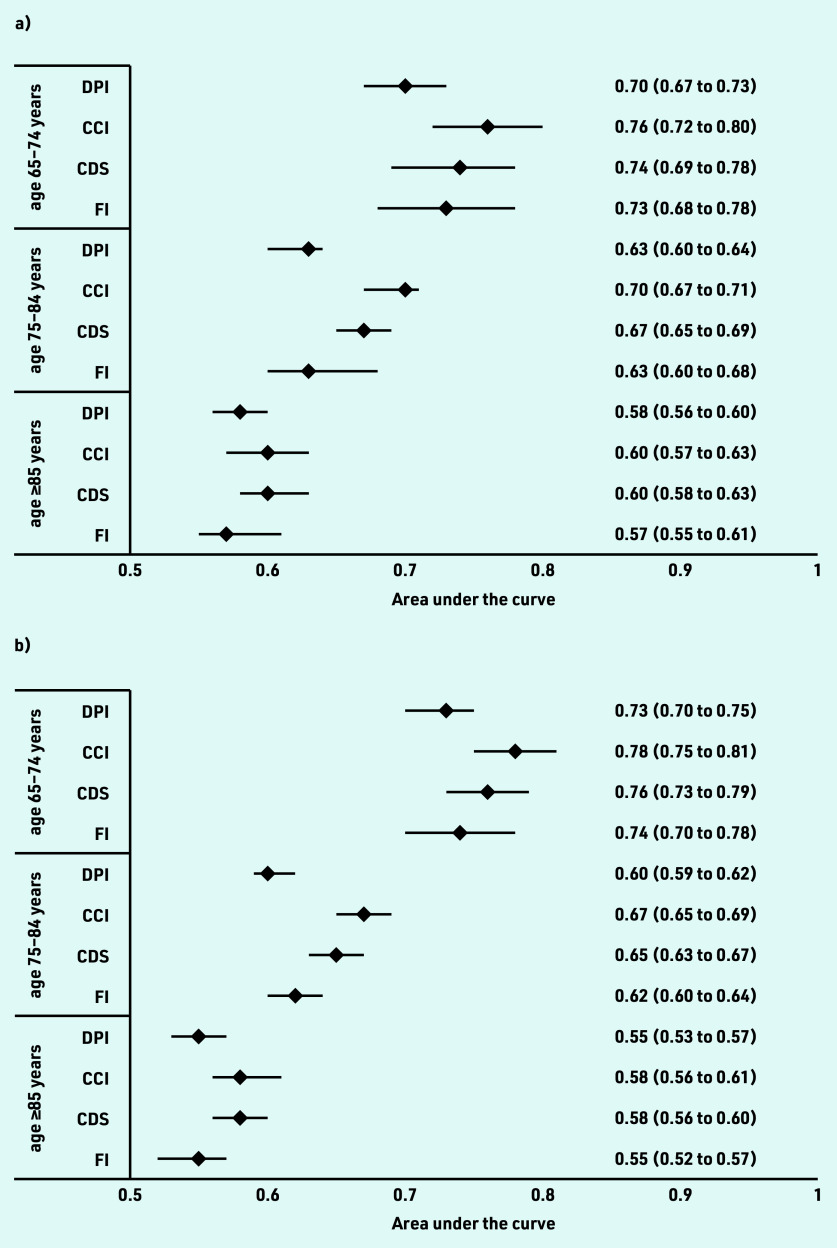
*Area under the receiver operating characteristic curve (AUC) for each index according to age group (65–74 years, top; 75–84 years, middle; ≥85 years, bottom) in (a) males (b) and females. CCI = Charlson Comorbidity Index. CDS = Chronic Disease Score. DPI = Dutch Polypharmacy Index. FI = Frailty Index.*

The performance of the four frailty measures showed different results when applied in subgroups of age categories and sex (Figure 1). The ability of the measures to discriminate between frail and non-frail decreased with increasing age. In patients aged 65–74 years, the AUCs ranged from 0.70 to 0.76 in males and 0.73 to 0.78 in females. In the 75–84 age group, the AUCs decreased to 0.63 to 0.70 in males and 0.60 to 0.67 in females. In the oldest age groups (≥85 years), the ability to identify frailty further decreased in males (0.57 to 0.60) and females (0.55 to 0.58). In all age groups, the CCI showed the most favourable results.

The diagnostic performance of the four measures was expressed as sensitivity, specificity, PPV, and NPV, with calculations based on the optimal cut-off value specific for each subgroup. Overall, the four measures showed poorer performance in males and females aged ≥85 years than younger age groups (Tables 3 and 4, respectively). A large decrease in the negative predictive value was seen in the oldest age group, which was more pronounced in females than in males, meaning that a larger proportion of patients classified as non-frail by the measures were considered frail according to the GP.

**Table 3.** Diagnostic performance of the four measures identify frailty according to age groups in males^a^

**Age group, years and measure**	**Cut-off**	**Sensitivity**	**Specificity**	**PPV**	**NPV**
**Age 65–74 (*n* = 8422)**					
DPI	1	0.76 (0.68–0.82)	0.65 (0.64–0.66)	0.03 (0.03–0.04)	0.99 (0.99–1.00)
CCI	6	0.60 (0.51–0.68)	0.82 (0.81–0.82)	0.05 (0.04–0.06)	0.99 (0.99–0.99)
CDS	7	0.70 (0.61–0.77)	0.67 (0.66–0.68)	0.03 (0.03–0.04)	0.99 (0.99–0.99)
FI	0.30	0.59 (0.50–0.67)	0.79 (0.79–0.80)	0.05 (0.04–0.06)	0.99 (0.98–0.99)

**Age 75–84 (*n* = 4529)**					
DPI	1	0.76 (0.72–0.80)	0.49 (0.47–0.51)	0.17 (0.16–0.19)	0.94 (0.92–0.95)
CCI	6	0.76 (0.73–0.80)	0.52 (0.51–0.54)	0.18 (0.17–0.20)	0.94 (0.93–0.95)
CDS	8	0.62 (0.58–0.66)	0.65 (0.64–0.67)	0.20 (0.18–0.22)	0.92 (0.91–0.93)
FI	0.30	0.57 (0.53–0.61)	0.65 (0.63–0.66)	0.19 (0.17–0.21)	0.91 (0.90–0.92)

**Age ≥85 (*n* = 1521)**					
DPI	1	0.74 (0.70–0.77)	0.42 (0.39–0.46)	0.45 (0.42–0.49)	0.71 (0.67–0.75)
CCI	8	0.53 (0.49–0.57)	0.62 (0.59–0.65)	0.47 (0.44–0.51)	0.67 (0.64–0.70)
CDS	5	0.85 (0.82–0.88)	0.29 (0.26–0.32)	0.44 (0.41–0.47)	0.75 (0.70–0.79)
FI	0.26	0.69 (0.65–0.73)	0.45 (0.41–0.48)	0.45 (0.42–0.48)	0.69 (0.65–0.73)

a *Cut-offs for identifying frailty (patients were considered frail above the cut-off) were based on the most optimal predicted probability estimated by the Youden index. CCI = Charlson Comorbidity Index. CDS = Chronic Disease Score. DPI = Dutch Polypharmacy Index. FI = Frailty Index. NPV = negative predictive value. PPV = positive predictive value.*

**Table 4.** Diagnostic performance of the four measures identify frailty according to age groups in females^a^

**Age group, years and measure**	**Cut-off**	**Sensitivity**	**Specificity**	**PPV**	**NPV**
**Age 65–74 (*n* = 8778)**					
DPI	1	0.80 (0.74–0.85)	0.65 (0.64–0.66)	0.05 (0.05–0.06)	0.99 (0.99–0.99)
CCI	5	0.74 (0.68–0.80)	0.70 (0.69–0.71)	0.06 (0.05–0.07)	0.99 (0.99–0.99)
CDS	8	0.63 (0.57–0.70)	0.78 (0.77–0.78)	0.07 (0.06–0.08)	0.99 (0.99–0.99)
FI	0.30	0.66 (0.59–0.72)	0.75 (0.74–0.76)	0.06 (0.05–0.07)	0.99 (0.99–0.99)

**Age 75–84 (*n* = 5609)**					
DPI	1	0.71 (0.68–0.74)	0.50 (0.48–0.51)	0.22 (0.20–0.23)	0.90 (0.89–0.91)
CCI	6	0.69 (0.66–0.72)	0.56 (0.55–0.58)	0.24 (0.22–0.25)	0.90 (0.89–0.91)
CDS	8	0.55 (0.52–0.58)	0.67 (0.65–0.68)	0.24 (0.23–0.26)	0.88 (0.87–0.89)
FI	0.30	0.58 (0.55–0.62)	0.62 (0.61–0.64)	0.23 (0.22–0.25)	0.88 (0.87–0.90)

**Age ≥85 (*n* = 2652)**					
DPI	1	0.68 (0.66–0.71)	0.41 (0.39–0.44)	0.53 (0.50–0.55)	0.58 (0.54–0.61)
CCI	8	0.44 (0.42–0.47)	0.69 (0.66–0.71)	0.58 (0.55–0.61)	0.56 (0.54–0.59)
CDS	7	0.60 (0.57–0.62)	0.51 (0.49–0.54)	0.54 (0.51–0.57)	0.57 (0.54–0.60)
FI	0.34	0.38 (0.35–0.41)	0.71 (0.69–0.73)	0.56 (0.52–0.59)	0.54 (0.52–0.57)

a *Cut-offs for identifying frailty (patients were considered frail above the cut-off) were based on the most optimal predicted probability estimated by the Youden index. CCI = Charlson Comorbidity Index. CDS = Chronic Disease Score. DPI = Dutch Polypharmacy Index. FI = Frailty Index. NPV = negative predictive value. PPV = positive predictive value.*

### Sensitivity analyses

When the performance of the frailty measures was calculated for each GP separately, the performance was again best for the CCI, and ranged from AUC 0.71 (95% CI = 0.65 to 0.78) to AUC 0.88 (95% CI = 0.83 to 0.93) (data not shown in table). For the majority of the GPs, the performance decreased with the older age subgroup, with an AUC below 0.7 in the oldest age group, in approximately 90% of the GPs for the four frailty measures.

The median age- and sex-standardised prevalence of frailty per GP practice, as classified by the GP, was 11.0% (IQR 9.5–14.0). The performance of the frailty measures was tested when only including GP practices with age- and sex-standardised frailty prevalence within the IQR. The measures’ performance was similar compared with the total population (Supplementary Figure S2).

## DISCUSSION

### Summary

This study investigated the diagnostic performance of the DPI, CCI, CDS, and FI for the identification of frail older adults with the use of electronic medical records of GPs compared with the clinical judgement of GPs. An acceptable performance, based on the AUC, was found for the CCI in the total sample, and a poor performance for the DPI, CDS, and FI. When stratifying the results according to age, the diagnostic performance was acceptable for all indexes in the youngest age group (65–74 years). However, the performance decreased for the higher age groups, showing a poor to failed performance in patients aged ≥85 years, and worse performance in females than males.

### Strength and limitations

A limitation of the current study includes the clinical judgement of the GP as a dichotomous definition, thereby ignoring the complexity of frailty. The use of more than two frailty categories has been suggested. For instance, the electronic FI as implemented in the UK uses four frailty categories.^13^ A strength of the current study was the use of GPs’ clinical judgement as a reference standard. Most previous studies validated the FI by prognostically reporting associations with future adverse events or based on statistical distributions. Although their judgement of frailty will show within- and between-GP variation, the GPs’ judgement on the presence and absence of frailty was found to be the best predictor of mortality.^10^ The sensitivity analysis among GP practices with a frailty prevalence within the IQR showed that the AUCs were similar compared with the AUCs in the total population. This indicates that the indexes were robust. Furthermore, it should be noted that data were derived from GP practices that routinely coded frailty as part of older care programmes. This ensures that frailty was registered. Another strength was the large sample size that made it possible to stratify the population to compare the performance of the frailty measures across sex and age groups.

### Comparison with existing literature

A previous study that evaluated a polypharmacy score and the FI in a primary care sample against Fried’s frailty criteria and clinical judgement by an expert panel showed similar performance as observed in the current study.^3^^,^^17^ Furthermore, in a systematic review, the psychometric properties of various frailty measures were investigated, and an association between the FI and several adverse health outcomes was consistently present. However, the ability of the measures to discriminate between people who will experience such an event and those who will not was poor to moderate, with the lowest AUCs in studies consisting of relatively older people.^22^^–^24 Adjustment for age and sex and consultation gap resulted in an improved AUC.^22^

The performance of the multimorbidity-driven measures decreased with increasing age. An explanation might be that, with increasing age, a survival bias of people with relatively few comorbidities occurs.^25^ This was reflected by a larger proportion of the patients being classified as non-frail by the measures while considered frail by the GP in the oldest age group. In these patients, other factors then multimorbidity may be more important in deciding whether patients are judged as being frail. When classifying patients’ frailty status, GPs typically use a broader definition, also taking into account functional, cognitive, emotional, and social aspects, and the type and number of complications and medicines.^9^^,^^26^^,^^27^ Characteristics related to more sudden changes might be useful to consider specifically, such as acute hospital admissions, falls, or more specific conditions such as dementia, forgetfulness, or incontinence.

Regarding the observed gender differences, previous studies already showed that females accumulate more deficits,^23^^,^^26^^,^^27^ resulting in higher scores on a comorbidity index than males. Despite this higher proportion of deficits, the risk of mortality in females is lower than in males because of the higher tolerability of deficits in females, specifically at an older age.^28^ This may call for the development of sex-specific cut-off scores to increase the diagnostic accuracy of measures.

### Implications for research and practice

The results suggest that the current electronically derived measures of frailty are applicable for identifying frailty in individuals up to a certain age in clinical practice and research. However, for the oldest old, it may be necessary to consider additional information to identify frail people.

Future research should explore factors beyond multimorbidity measures, such as characteristics related to more sudden changes including acute hospital admissions, falls, or specific conditions such as dementia, forgetfulness, or incontinence.

To advance research in this field, it would be beneficial to combine data that are available in GP practices with more comprehensive data sources. For example, data from nursing homes could provide valuable additional insights.

## Data

The dataset generated and analysed during the current study are not publicly available but are available from the corresponding author on reasonable request and after approval of the Compliance Committee of Stichting Informatievoorziening voor Zorg en Onderzoek.
